# Minimally Invasive Reconstruction of Vertebral Arch in Spondylolisthesis in Children and Adolescents

**DOI:** 10.17691/stm2021.13.5.08

**Published:** 2021-10-29

**Authors:** А.R. Syundyukov, N.S. Nikolayev, V.А. Kuzmina, S.А. Aleksandrov, P.N. Kornyakov, V.Yu. Emelyanov

**Affiliations:** Head of the Pediatric Traumatological and Orthopedic Unit Federal Center of Traumatology, Orthopedics and Arthroplasty, Ministry of Health of the Russian Federation, 33 Fedor Gladkov St., Cheboksary, Chuvash Republic, 428020, Russia; Professor, Chief Doctor Federal Center of Traumatology, Orthopedics and Arthroplasty, Ministry of Health of the Russian Federation, 33 Fedor Gladkov St., Cheboksary, Chuvash Republic, 428020, Russia;; Head of the Department of Traumatology, Orthopedics and Extreme Medicine Chuvash State University named after I.N. Ulyanov, 15 Moskovsky Prospect, Cheboksary, Chuvash Republic, 428015, Russia; Functional Diagnostician Federal Center of Traumatology, Orthopedics and Arthroplasty, Ministry of Health of the Russian Federation, 33 Fedor Gladkov St., Cheboksary, Chuvash Republic, 428020, Russia; Traumatologist-Orthopedist Federal Center of Traumatology, Orthopedics and Arthroplasty, Ministry of Health of the Russian Federation, 33 Fedor Gladkov St., Cheboksary, Chuvash Republic, 428020, Russia; Traumatologist-Orthopedist Federal Center of Traumatology, Orthopedics and Arthroplasty, Ministry of Health of the Russian Federation, 33 Fedor Gladkov St., Cheboksary, Chuvash Republic, 428020, Russia; Researcher Federal Center of Traumatology, Orthopedics and Arthroplasty, Ministry of Health of the Russian Federation, 33 Fedor Gladkov St., Cheboksary, Chuvash Republic, 428020, Russia;; Associate Professor Chuvash State University named after I.N. Ulyanov, 15 Moskovsky Prospect, Cheboksary, Chuvash Republic, 428015, Russia

**Keywords:** spondylolisthesis, isthmic spondylolisthesis, spondylodesis, vertebral arch reconstruction

## Abstract

**The aim of the study** was to assess the effectiveness of the minimally invasive technique used to reconstruct the vertebral arch with a pedicle screw hook system in grade I isthmic spondylolisthesis in comparison with the traditional technique of segment stabilization with interbody fusion.

**Materials and Methods:**

The study included 26 patients aged from 11 to 17 years. The follow-up period lasted from 1 to 7 years. Two groups were formed: in group 1 (n=6), segments L_5_–S_1_ were stabilized using the traditional technique; in group 2 (n=20), the arch of the L_5_ vertebra was reconstructed by means of minimally invasive surgery. The pain syndrome was assessed in each study group using the visual analogue scale and Macnab criteria before and after surgery; blood loss, duration of surgery in minutes, and hospitalization in days were also measured.

**Results:**

According to the Macnab scale, the two presented techniques did not show any statistically significant differences; however, when the arch synthesis technique was employed the spinal motion segment remained intact. Furthermore, in group 2, the volume of blood loss was smaller (44.0±19.6 compared to 300.0±130.4 ml, p<0.0001), the duration of the operation was 176.0±41.6 compared to 349.2±93.2 min, p<0.0001, and hospital stay was 6.9±1.6 compared to 10.0±2.1 days, p=0.0025 in the control group.

**Conclusion:**

The technique of vertebral arch reconstruction by a minimally invasive access gives the possibility to stabilize the spinal motion segment and to preserve biomechanics and movements in the spine. This technique allows for shorter inpatient stays for patients as well as earlier recovery and rehabilitation due to reduced surgery time and blood loss.

## Introduction

The main cause of spondylolisthesis and its progression in children and adolescents is tissue tropism disorders and microtraumas [1, 2] resulting in the appearance of lysis zones in the interarticular part of the vertebral arch. Spondylolisthesis leads to instability and abnormal interrelationships of the vertebrae resulting in turn in sagittal imbalance, marked pain syndrome in the back which may irradiate to the legs and ultimately impair the function of lower limbs [3]. The zones of lysis are localized on the L_5_ vertebra in 80% of cases, more rarely at the level of the L_4_ vertebra, localizations of this pathology at other levels of the lumbar spine as well as multilevel injuries have been also described [4].

The classic method of treating spondylolysis and spondylolisthesis is surgical intervention included decompression, reduction of the slipped vertebra, transpedicular fixation of the spine segments, and creation of 360° spondylodesis [5–8]. In case of spondylolysis or grade I spondylolisthesis in children and adolescents with a pain syndrome but without neurological disturbances, the main approach is conservative therapy, although an alternative surgical way of treatment is also known. The so-called surgical reconstruction of the vertebral arch suggested by Buck in 1970 [9] has been modified and described in the works of a number of other authors [10, 11]. It makes it possible to stabilize the segment and prevents the progression of slippage without its fixation. The main point of all proposed methods is to create prerequisites for the consolidation of the vertebral arch. And the fixation techniques within one vertebra vary from the compression with a screw to fastening with a wire. With the development of minimally invasive techniques, a low-traumatic approach to this type of surgical intervention has been also devised [12].

**Figure 1.**
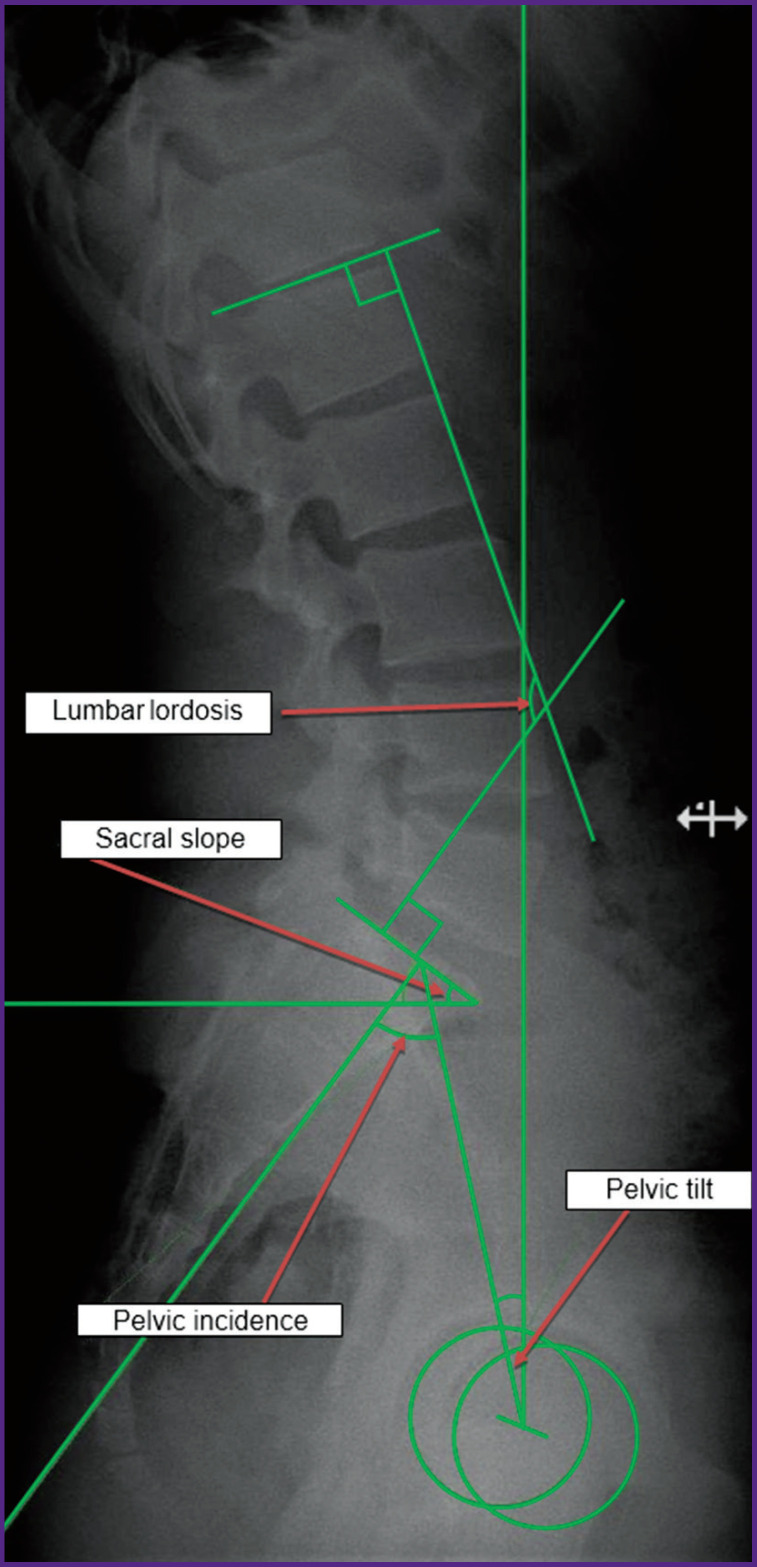
Angles of spinopelvic interrelationship (pelvic incidence, pelvic tilt, sacral slope, lumbar lordosis) on the radiograph

The emergence of the lysis zones in the interarticular part of the arch is connected with the load on this spine segment: at the pivot point (the middle osteoligamentous column between the last lumbar and the first sacral vertebrae) the maximal stress on the spine is concentrated [13]. Abnormal changes in the spinopelvic parameters of the sagittal balance may cause this pathology [14]. Despite the modern classification of spondylolisthesis types based on the key angle values of the spinopelvic relationships such as pelvic incidence — PI, pelvic tilt — PT, sacral slope — SS, lumbar lordosis — LL (Figure 1), the question of standard parameters for the pelvis and spine in spondylolysis and low-grade spondylolisthesis in children and adolescents and their changes after the operation has not been fully resolved [15]. Data on the effectiveness of the mini-invasive vertebral arch reconstruction with a pedicle screw hook construct (according to Morscher) are also insufficient in the literature and publications on the application of intraoperative neuromonitoring during such interventions are completely absent.

**The aim of the study** was to assess the effectiveness of the vertebral arch reconstruction with a pedicle screw hook system in grade I isthmic spondylolisthesis in children and adolescents using minimally invasive surgery to prevent spondylolisthesis progression and restore normal lordosis parameters in comparison with the traditional technique of segment fixation.

## Materials and Methods

The study included 26 patients (9 females and 17 males) aged from 11 to 17 years with spondylolysis and low-grade isthmic spondylolisthesis followed-up from 2010 to 2017. The study was conducted in compliance with the Declaration of Helsinki (2013) and approved by the Ethical Committee of the Federal Center of Traumatology, Orthopedics and Arthroplasty (Cheboksary, Russia). Informed written consent was obtained from patients over 15 years old and from the parents of children under 15 years following the Federal Law “Fundamentals of the Legislation of the Russian Federation on the Protection of Citizens’ Health” (2011).

The pathology was localized on the L_5_ vertebra in all participants of the study, besides, neurological disorders, which included persistent pain syndrome resistant to conservative treatment, were absent. Two groups were formed: group 1 (n=6) was retrospective control group in which patients underwent traditional operation for segment L_5_–S_1_ stabilization with a transpedicular screw system in combination with interbody spondylodesis from the open access; group 2 (n=20) represented a prospective group in which the reconstruction of the L_5_ arch was performed with a pedicle screw hook system using minimally invasive technique which consisted in consolidation of the interarticular part of the arch and stabilization of the spinal motion segment using its own articular segment. All patients in both groups had grade I spondylolisthesis according to the Meyerding classification since the application of the pedicle screw hook construct is designed to be used only at this grade. The follow-up period covered from 1 to 7 years.

In group 1, the traditional variant of intervention was used: after skin incision, the dorsal compartments of the spine and sacrum were skeletonized in the zone of the proposed spondylodesis. Then, transpedicular screws were installed into the arches of the L_5_ and S_1_ vertebrae, resection of the inferior articular processes of the L_5_ vertebra and superior articular processes of the S_1_ vertebra was made, the moveable arch of the L_5_ vertebra was partly resected, and discectomy with the interbody cage installation was performed. At the next step, the rods were inserted into the screw heads and a reduction maneuver was done (in case of spondylolisthesis) fixing the rods in the screws. At the final step, the operative wound was sutured in layers [15].

**Figure 2.**
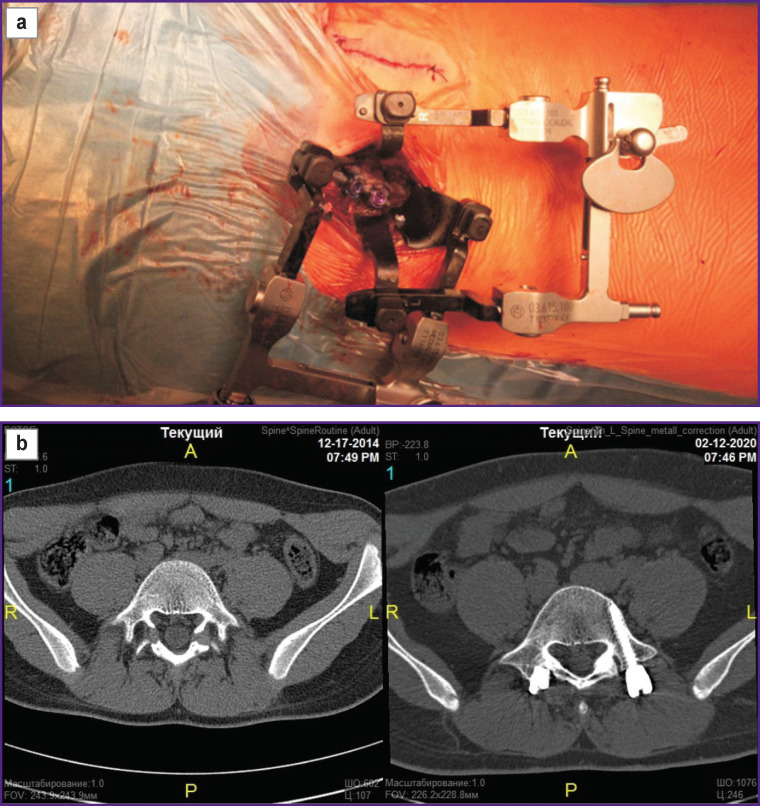

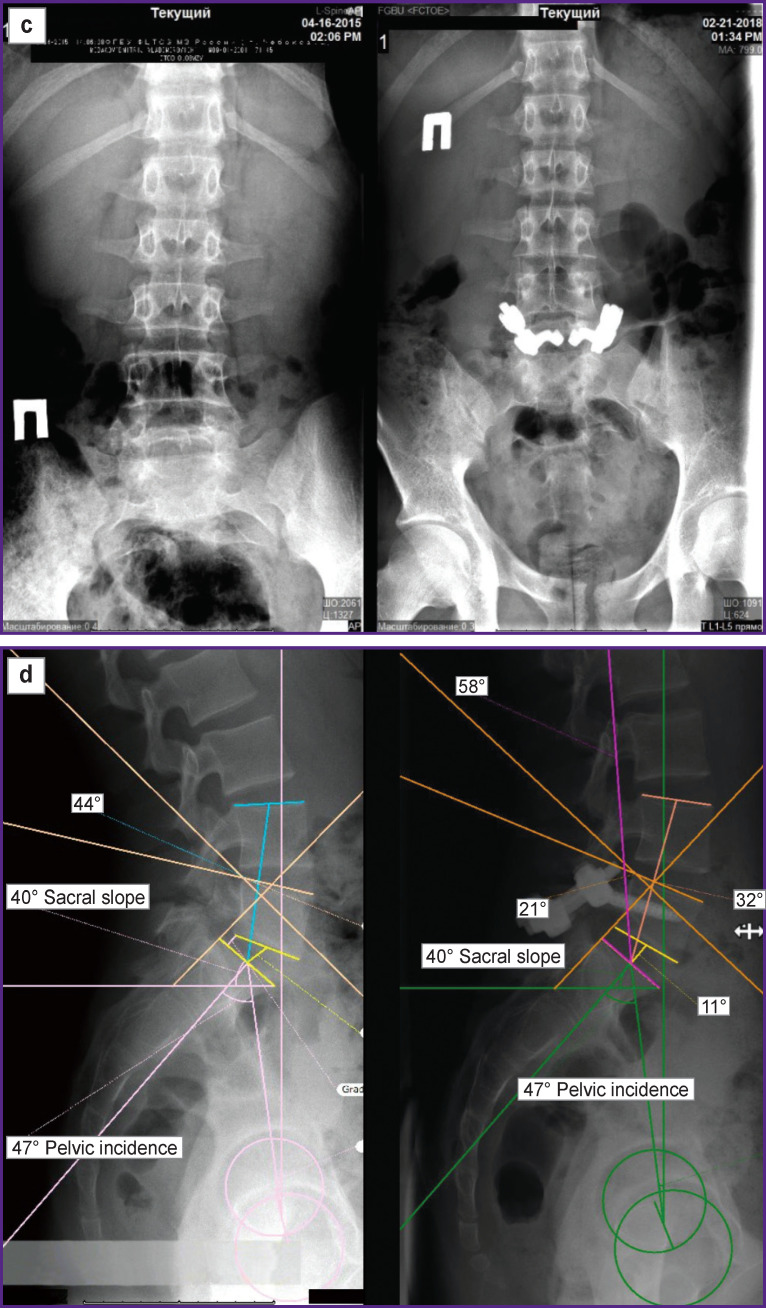

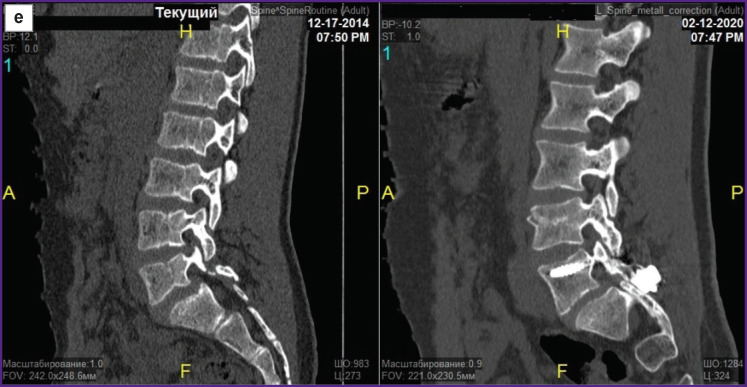
Clinical example of treating spondylolisthesis using minimally invasive technique: (а) minimally invasive dorsal access at the level of the L_5_ vertebra; (b) axial computed tomography: left — preoperative, shows the defect at the level of the L_5_ vertebra; right — postoperative, shows transpedicular screws in the arch of the L_5_ vertebra; (c) radiograph in the direct anterior projection: left — preoperative, shows the defect at the level of the L_5_ vertebra; right — postoperative, shows defect fixation with transpedicular screws at the level of the L_5_ vertebra; (d) radiograph in the lateral projection: left — preoperative, shows the defect at the level of the L_5_ vertebra; right — postoperative, defect was removed using pedicle-screw and hook constructs; (e) lateral CT: left — preoperative, shows the defect at the level of the L_5_ vertebra; right — postoperative — shows two-sided screws on the pedicle and rod and hook constructs on the L_5_ vertebra for defect correction

In group 2, the following tactics was used to surgically reconstruct the L_5_ arch: under the control of the electro-optical transducer (EOT), landmarks of the proposed incisions were applied on the skin of the patient lying in a prone position under general anesthesia, incisions up to 2.5 cm in length were made in soft tissues paravertebrally in the region of the L_5_ vertebra, a petal telescopic paravertebral dilator was installed. Access to the dorsal elements of the L_5_ vertebra was achieved by muscle separation and preservation of the supra- and interspinal ligaments. The zone of spondylolysis was treated with curettes and a drill. This stage is technically difficult and important, as the lysis zone is anatomically located over the site of the root exit, therefore, there is a risk of moving the instrument downward and medially and damaging the nerve structures. Further, a synthetic bone transplant in the form of a paste was injected into the lysis zone; two supralaminar hooks with a displaced working body were placed under the arch of the L_5_ vertebra; a transpedicular cannulated screw was mounted along the guiding wires under the EOT control using a minimally invasive technique. A rod was installed, fastened with nuts in the element heads, and compression was performed. The wound was sutured. The procedure was done in the same succession from the opposite side (Figure 2).

This method makes it possible to create conditions for vertebral arch consolidation, to make the reduction acceptable, and, at the same time, to preserve the mobility of the spinal motion segment [16].

In the postoperative period, patients were verticalized on the first day after the surgical intervention and 3‒5 days later were discharged for the outpatient treatment. All examined patients underwent radiography before and after the operation to assess PI, PT, SS, LL. The results of the study were evaluated using the subjective assessment Macnab scale [17], visual analogue pain scale (VAS) before and after the operation. Reference values for lordosis were calculated for each patient using the following formula: L=0.62**·**PI+27.61 [18]. Functional results were assessed by clinical data (restrictions of the spine motion and bending) and MRT data (intervertebral disc height in standing and presence of degeneration signs).

**Study limitations:** small sample, unicenter study, relatively short follow-up time, absence of the radiographic control of the operated segment mobility.

**Statistical data** were processed using GraphPad Prism 8 software. Descriptive statistics were chosen according to the algorithm as described before [19]. Normal distribution of the samples was tested using Kolmogorov–Smirnov criterion; in our case, normal distribution was noted in the majority of the groups. The arithmetic mean (M) and standard deviation (SD) were calculated for each parameter. Percentage (P) and its standard deviation (σ_р_) were calculated for the qualitative data. Statistical significance of value differences was assessed using Mann–Whitney test, p<0.05 was taken as critical.

## Results

In both groups, spondylolisthesis was fully corrected.

The follow-up period lasted from 1 to 7 years. Assessing the results of observation in the period from 3 months to 4 years it has been established that there were no statistically significant differences in the values obtained by the Macnab scale between the two presented techniques, however, the spine motion segment remained intact in case of using the vertebral arch synthesis technique.

Very good results by the Macnab scale were noted in 4 patients from the group 1 with stabilization of L_5_‒S_1_ segment, in 2 patients, the results were good (there remained complaints of the periodic pains after the loads which did not require analgetics and did not interfere with their everyday activities).

Very good results by the Macnab scale were achieved in 17 patients from group 2 who underwent arch reconstruction. Pains according to VAS were completely arrested. Two patients complained of the periodic pains which resolved a year after the operation. Persistent pain syndrome was preserved in one patient. One more patient was noted to have ligature fistulas, which closed after ligature removal. This problem was interpreted by us as an individual reaction to the suture material (Table 1).

**Table 1:** Indices of treating spondylolisthesis with different techniques

Parameters	Group 1	Group 2	p
Patient number (male/female)	6 (4/2)	20 (13/7)	—
Assessment by VAS, M±SD:			
before operation	2.50±0.50	2.95±0.80	0.3175
after operation	1.0±0.60	0.65±0.60	0.2618
Results by Macnab scale (%), P±σ_P_:			
excellent	66.70±19.0	80.0±9.0	
good	33.30±19.0	20.0±9.0	
Blood loss (ml), M±SD	300.0±130.4	44.0±19.6	<0.0001
Duration of the operation (min), M±SD	349.2±93.2	176.0±41.6	<0.0001
Hospital stay (days), M±SD	10.0±2.1	6.9±1.6	0.0025

Functional results were assessed by the clinical data (motion and bending restrictions in the spine were not observed) and by the MRT data (the height of the intervertebral discs was preserved, degeneration signs were absent). 11 patients from the arch reconstruction group returned to their physical exercise and sports.

Interbody spondylodesis has formed in all patients undergone the traditional operation. In the group with the L_5_ arch reconstruction, the signs of the arch consolidation according to CT performed 3 months and 1 year after the operation were noted in 14 patients. Partial consolidation on one side was observed in 3 patients, and in 3 more patients, fusion of the vertebral arch segments was absent: in 2 of them, the achieved correction and stability of the metal construction were preserved without diastasis enlargement; despite the construction stability and absence of lysis zone expansion, in 1 patient, there was progressive displacement of the vertebra by 5 mm and pain syndrome intensification which required repeat operation using the traditional technique of segment fixation.

Neuromonitoring was employed at all stages of the surgical intervention. Neuromonitoring data have shown that there was no approaching to the nerve structures during channel formation for transpedicular supporting elements and screw placement. This is confirmed by the correct placement of the transpedicular supporting elements assessed by the CT data: all transpedicular screws were installed correctly according to the Gerzbein scale. Absence of radicular symptoms after surgical treatment also speaks of the effectiveness of intraoperative monitoring at all stages of the operation.

Comparing the blood loss volume and hospital stays after the operation, the following tendency has been found: in case of minimally invasive method of the vertebral arch synthesis, the average blood loss was 6 times less and the hospital stay was 3 days shorter (Table 2).

**Table 2:** Indices of spinopelvic interrelationships in patients of both groups before and after the operation

Parameter	Group 1		Group 2		p
	M±SD	95% CI	M±SD	95% CI	
Pi	62.3±12.6	73.0–57.8	58.1±7.1	63.0–50.5	0.4475
Reference lordosis indices	66.3±7.8	68.8–60.6	63.7±4.4	66.2–61.4	0.4475
LL:					
before operation	73.0±9.4	77.0–67.0	61.1±9.0	67.0–58.0	0.0098
after operation	63.2±10.6	67.3–55.8	55.3±8.8	61.0–51.0	0.1185
SS:					
before operation	50.2±7.3	53.0–45.0	46.3±7.0	50.0–42.0	0.361
after operation	45.8±9.2	50.0–42.5	41.8±7.5	46.8–35.0	0.3626
PT:					
before operation	12.3±8.5	28.0–7.0	11.9±5.6	14.5–5.0	0.8703
after operation	15.2±9.7	25.5–10.0	16.0±6.9	19.8–11.0	0.6667

When assessing spinopelvic relationship, no marked deficit of the lumbar lordosis was found in both groups, there was also noted that pelvic parameters change statistically significantly after the operation towards the reference lordosis values. The relatively balanced values before the operation in patients of both groups are, to our opinion, associated with an adequate work of the compensatory mechanisms of the body in low-grade spondylolysis and spondylolisthesis. Despite the absence of the segment fixation and manipulations directed to lordosis formation by means of interbody cage in group 2, positive changes of the indices after the operation were noted in the patients of this group (see Table 2).

## Discussion

At present, there are several known modifications of mini-invasive methods of treating spondylolisthesis including those with segment fixation which are used in the surgery of isthmic spondylolysis with spondylolisthesis in adults. These methods include segment fixation with transforaminal lumbar interbody spondylodesis with direct muscle-sparing decompensation of a part of the defect [20, 21]. The mini-invasive technique employed in this study consists in consolidation of the interarticular part of the vertebral arch with stabilization of the spinal motion segment using its own articular process. This operation is relatively new; therefore, the problems and nuances of its performance are being widely discussed [12, 22]. The application of the given reconstruction technique allowed us to create conditions for consolidation of the vertebral arch, to realize an acceptable reduction, and to preserve mobility in the spinal motion segment [16].

The data obtained in our work have demonstrated that no changes of the lumbar lordosis relative pelvic parameters in spondylolysis and low-grade spondylolisthesis are observed and, therefore, there is no need to correct these parameters.

Positive changes of the radiological parameters after vertebral arch osteosynthesis, vertebra reduction, absence of disease progression, and reliable elimination of the pain syndrome allow us to consider this operation to be a worthy alternative to the classic method which is comparable with the data obtained by other investigators on adult patients [23, 24]. Indices of intraoperative blood loss, returning to sports activities of the majority of patients, decrease in hospital bed days, and, consequently, hospital expenses make this intervention the method of choice in this pathology. The use of similar operative technique on children with formation of spondylodesis gave the same results in regard to blood loss speaking of the representativeness of the data obtained by us [25].

Neuromonitoring at the stage of the resection of the lysed part of the arch is explained by the necessity of complete and safe treatment of the lysis zone. As the zone of spondylolysis is anatomically located over the lace of the nerve root exit, there exists a risk of moving the instrument lower and medially and damaging the nerve structures. The correctness of the transpedicular screw installation and absence of radicular symptoms after surgical treatment indicate the effectiveness of intraoperative neuromonitoring at all stages of the operation.

## Conclusion

The technique of reconstructing the L_5_ arch from minimally invasive access using transpedicular cannulated screws for treatment of spondylolisthesis in children and adolescents gives the possibility to stabilize the spinal motion segment, reliably remove the pain syndrome preserving, at the same time, biomechanics and movements in the spine. This technique allows for shorter inpatient stays for patients as well as earlier recovery and rehabilitation.
